# Supplementary material for: Maternal obesity increases hypothalamic miR-505-5p expression in mouse offspring leading to altered fatty acid sensing and increased intake of high-fat food
Source: PLoS Biol. 2024 Jun 4;22(6):e3002641. doi: 10.1371/journal.pbio.3002641 (PMC11149872; doi:10.1371/journal.pbio.3002641)
Supplement: S1 Table — (A) Primer sequences of primers used in SYBR qPCR purchased from Sigma Aldrich. (B) Taqman Assay IDs of probes used in Taqman qPCR purchased from Thermofisher Scientific. (C) miRCURY LNA miRNA PCR assays purchased from Qiagen. (DOCX) [file pbio.3002641.s001.docx]

| Gene | Forward (5’-3’) | Reverse (5’-3’) |
| --- | --- | --- |
| Acot9 | gcgatacggctttggacctt | tccagactgtggatacgcct |
| Agpat3 | ctcacccctcttcagcttcg | gactccgaaggaagctgctc |
| Sdha | tcgacaggggaatggtttgg | taatcttccctggcatgggc |
| Slc25a10 | gcgggactacatgaccaagg | agagtagttgcgtcgttggc |
| Slc27a4 | cagccgggtcacaatgctac | tgaagacccggatgaaacgc |

A

B

| Gene | Assay ID |
| --- | --- |
| Atp11c | Mm01297974_m1 |
| Cpt1a | Mm01231183_m1 |
| Soat1 | Mm00486279_m1 |
| Sdha | Mm01352366_m1 |

C

| Gene | Assay ID |
| --- | --- |
| miR-505-5p | YP02104807 |
| Snord68 | YP00203911 |
| USP6 | YP02119464 |

**Supplementary Table 1:**

A) Primer sequences of primers used in SYBR qPCR purchased from Sigma Aldrich B) Taqman Assay IDs of probes used in Taqman qPCR purchased from Thermofisher Scientific C) miRCURY LNA miRNA PCR assays purchased from Qiagen.
