# Supplementary material for: Maternal obesity increases hypothalamic miR-505-5p expression in mouse offspring leading to altered fatty acid sensing and increased intake of high-fat food
Source: PLoS Biol. 2024 Jun 4;22(6):e3002641. doi: 10.1371/journal.pbio.3002641 (PMC11149872; doi:10.1371/journal.pbio.3002641)
Supplement: S2 Table — (DOCX) [file pbio.3002641.s002.docx]

|  | logFC | PValue |
| --- | --- | --- |
| mmu-miR-505-5p | 2.26790985 | 0.0101714 |
| mmu-let-7e-5p | 1.49047862 | 0.02151258 |
| mmu-let-7k | 1.418607 | 0.02855474 |
| mmu-miR-92b-5p | 1.01339219 | 0.04323239 |

**Supplementary Table 2:**

Significantly regulated miRNAs detected in paraventricular nucleus of the hypothalamus of offspring from obese mothers in miR-sequencing analysis
