# Supplementary material for: Maternal obesity increases hypothalamic miR-505-5p expression in mouse offspring leading to altered fatty acid sensing and increased intake of high-fat food
Source: PLoS Biol. 2024 Jun 4;22(6):e3002641. doi: 10.1371/journal.pbio.3002641 (PMC11149872; doi:10.1371/journal.pbio.3002641)
Supplement: S3 Table — (DOCX) [file pbio.3002641.s003.docx]

|  | logFC | PValue |
| --- | --- | --- |
| mmu-miR-505-5p | 3.15201921 | 7.38E-05 |
| mmu-miR-5105 | 1.30542539 | 0.00184717 |
| mmu-miR-6540-5p | 1.49630597 | 0.00273614 |
| mmu-let-7e-5p | 1.39679886 | 0.00330176 |
| mmu-miR-98-5p | 1.17038042 | 0.003708 |
| mmu-let-7f-5p | 1.26987087 | 0.00463843 |
| mmu-miR-6538 | 1.19286835 | 0.00473093 |
| mmu-let-7k | 1.31238993 | 0.00499318 |
| mmu-let-7c-5p | 1.29452908 | 0.00527431 |
| mmu-let-7d-5p | 1.37471051 | 0.00608837 |
| mmu-let-7a-5p | 1.21025834 | 0.008328 |
| mmu-miR-485-5p | 1.04037587 | 0.01619518 |
| mmu-miR-1224-3p | 1.10615633 | 0.02779869 |
| mmu-miR-383-5p | 1.1783129 | 0.03642917 |

**Supplementary Table 3:**

Significantly regulated miRNAs detected in arcuate nucleus of the hypothalamus of offspring from obese mothers in miR-sequencing analysis
