# Supplementary material for: Maternal obesity increases hypothalamic miR-505-5p expression in mouse offspring leading to altered fatty acid sensing and increased intake of high-fat food
Source: PLoS Biol. 2024 Jun 4;22(6):e3002641. doi: 10.1371/journal.pbio.3002641 (PMC11149872; doi:10.1371/journal.pbio.3002641)
Supplement: S4 Table — Gene; gene symbol for human orthologue of mir505 target, chr; chromosome where Gene is locates, Summary; summarised count of supporting evidence across the four analyses, MAGMA P-value; p-value from the gene-level MAGMA test, MAGMA FDR-corrected P-value; MAGMA P-value after FDR correction, GWAS Signal; rsid for the GWAS signal proximal to Gene, position; chromosomal position of GWAS signal (GRCh 37), a1; effect allele, a0; alternate allele, freq1; observed frequency of a1, beta1; effect size estimate per copy of a1, se; standard error of beta1, p; p-value for the association, Is closest?; is Gene the closest gene to GWAS Signal, N ABC Enhancers; number of known ABC enhancers within the LD window (R-sq>0.8) of the GWAS Signal, P SMR; p-value for the SMR test, FDR-corrected P SMR; P SMR after FDR correction, P HEIDI; p-value for the Heidi test. (DOCX) [file pbio.3002641.s004.docx]

| Gene | chr | Summary | BMI (UK Biobank + GIANT meta-analysis) | | | | | | | | | | | | | | |
| --- | --- | --- | --- | --- | --- | --- | --- | --- | --- | --- | --- | --- | --- | --- | --- | --- | --- |
|  |  |  | MAGMA P-value | MAGMA FDR-corrected P-value | GWAS Signal | position | a1 | a0 | freq1 | beta1 | se | p | Is closest? | N ABC Enhancers | P SMR | FDR-corrected P SMR | P HEIDI |
| CPT1A | 11 | 3 | 2.8E-05 | 2.7E-04 | rs497261 | 68192244 | T | C | 0.673 | 0.011 | 0.002 | 2.5E-08 | F | NA | 8.5E-06 | 2.1E-05 | 1.7E-03 |
| HSD17B12 | 11 | 3 | 3.2E-13 | 9.3E-12 | rs10838193 | 43898020 | G | A | 0.663 | 0.011 | 0.0018 | 1.7E-09 | F | 5 | 9.7E-26 | 4.9E-25 | 6.6E-04 |
| HINT1 | 5 | 3 | 9.7E-05 | 7.0E-04 | rs1363695 | 130378027 | C | T | 0.764 | 0.013 | 0.002 | 8.1E-10 | T | 1 | 8.4E-05 | 8.4E-05 | 2.0E-07 |
| SLC27A4 | 9 | 3 | 9.2E-07 | 1.3E-05 | rs7871866 | 131027982 | G | C | 0.840 | -0.018 | 0.0024 | 7.6E-14 | F | 19 | NA | NA | NA |
| SLC25A10 | 17 | 2 | 0.338 | 0.392 | rs11658335 | 80084821 | C | T | 0.418 | 0.011 | 0.0017 | 8.6E-11 | F | 17 | NA | NA | NA |
| EPHX1 | 1 | 2 | 3.6E-03 | 1.6E-02 | rs10915840 | 225668524 | G | A | 0.721 | 0.011 | 0.0019 | 1.9E-08 | F | NA | NA | NA | NA |
| HADHA | 2 | 2 | 0.666 | 0.689 | rs12468863 | 26940294 | C | T | 0.488 | 0.015 | 0.0016 | 6.6E-20 | F | 5 | NA | NA | NA |
| HSPA8 | 11 | 2 | 0.204 | 0.321 | rs7115089 | 122530591 | C | G | 0.628 | -0.011 | 0.0017 | 6.0E-11 | F | 4 | NA | NA | NA |
| AGPAT5 | 8 | 1 | 0.760 | 0.760 | NA | NA | NA | NA | NA | NA | NA | NA | NA | NA | 8.0E-05 | 8.4E-05 | 1.5E-02 |
| ERLIN1 | 10 | 1 | 0.055 | 0.146 | rs17094222 | 102395440 | T | C | 0.791 | -0.017 | 0.002 | 4.0E-18 | F | NA | NA | NA | NA |
| FDPS | 1 | 1 | 0.014 | 0.051 | NA | NA | NA | NA | NA | NA | NA | NA | NA | NA | 6.0E-05 | 8.4E-05 | 5.0E-02 |
| PITPNA | 17 | 1 | 0.276 | 0.348 | rs3923783 | 1843189 | C | A | 0.822 | 0.022 | 0.0022 | 4.1E-23 | F | NA | NA | NA | NA |
| ACAD9 | 3 | 1 | 0.172 | 0.295 | rs76594121 | 128189391 | T | G | 0.955 | 0.028 | 0.0047 | 1.6E-09 | F | NA | NA | NA | NA |
| ACADL | 2 | 1 | 0.019 | 0.062 | rs715 | 211543055 | T | C | 0.695 | -0.016 | 0.0019 | 1.3E-16 | F | NA | NA | NA | NA |
| AGPAT3 | 21 | 1 | 3.9E-03 | 1.6E-02 | NA | NA | NA | NA | NA | NA | NA | NA | NA | NA | NA | NA | NA |
| PAF1 | 19 | 1 | 1.8E-03 | 1.1E-02 | NA | NA | NA | NA | NA | NA | NA | NA | NA | NA | NA | NA | NA |
| DBI | 2 | 0 | 0.104 | 0.216 | NA | NA | NA | NA | NA | NA | NA | NA | NA | NA | NA | NA | NA |
| AGPAT4 | 6 | 0 | 0.524 | 0.563 | NA | NA | NA | NA | NA | NA | NA | NA | NA | NA | NA | NA | NA |
| HMGCL | 1 | 0 | 0.173 | 0.295 | NA | NA | NA | NA | NA | NA | NA | NA | NA | NA | NA | NA | NA |
| LPCAT3 | 12 | 0 | 0.267 | 0.348 | NA | NA | NA | NA | NA | NA | NA | NA | NA | NA | NA | NA | NA |
| CRAT | 9 | 0 | 0.076 | 0.178 | NA | NA | NA | NA | NA | NA | NA | NA | NA | NA | NA | NA | NA |
| ERLIN2 | 8 | 0 | 0.356 | 0.397 | NA | NA | NA | NA | NA | NA | NA | NA | NA | NA | NA | NA | NA |
| PTK2 | 8 | 0 | 0.080 | 0.178 | NA | NA | NA | NA | NA | NA | NA | NA | NA | NA | NA | NA | NA |
| SGPL1 | 10 | 0 | 0.222 | 0.321 | NA | NA | NA | NA | NA | NA | NA | NA | NA | NA | NA | NA | NA |
| SOD1 | 21 | 0 | 0.242 | 0.334 | NA | NA | NA | NA | NA | NA | NA | NA | NA | NA | NA | NA | NA |
| ACOT9 | X | 0 | NA | NA | NA | NA | NA | NA | NA | NA | NA | NA | NA | NA | NA | NA | NA |
| DHCR7 | 11 | 0 | 0.045 | 0.131 | NA | NA | NA | NA | NA | NA | NA | NA | NA | NA | NA | NA | NA |
| HADH | 4 | 0 | 0.338 | 0.392 | NA | NA | NA | NA | NA | NA | NA | NA | NA | NA | NA | NA | NA |
| PI4KA | 22 | 0 | 0.123 | 0.237 | NA | NA | NA | NA | NA | NA | NA | NA | NA | NA | NA | NA | NA |
| SOAT1 | 1 | 0 | 0.220 | 0.321 | NA | NA | NA | NA | NA | NA | NA | NA | NA | NA | NA | NA | NA |

**Supplementary Table 4:** Overlap of miR-505-5p targets with human genetic data on BMI variation and other functional datasets. Gene; gene symbol for human orthologue of mir505 target, chr; chromosome where Gene is locates, Summary; summarised count of supporting evidence across the four analyses, MAGMA P-value; p-value from the gene-level MAGMA test, MAGMA FDR-corrected P-value; MAGMA P-value after FDR correction, GWAS Signal; rsid for the GWAS signal proximal to Gene, position; chromosomal position of GWAS signal (GRCh 37), a1; effect allele, a0; alternate allele, freq1; observed frequency of a1, beta1; effect size estimate per copy of a1, se; standard error of beta1, p; p-value for the association, Is closest?; is Gene the closest gene to GWAS Signal, N ABC Enhancers; number of known ABC enhancers within the LD window (R-sq>0.8) of the GWAS Signal, P SMR; p-value for the SMR test, FDR-corrected P SMR; P SMR after FDR correction, P HEIDI; p-value for the HEIDI test.
